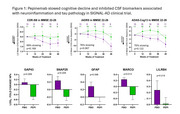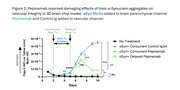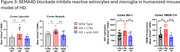# Targeting SEMA4D/PLXN signaling through reactive glia as a common pathology for the treatment of neurodegenerative disorders

**DOI:** 10.1002/alz70859_103679

**Published:** 2025-12-25

**Authors:** Elizabeth E Evans, Terrence L Fisher, Vikas Mishra, Leslie Balch, Elaine E Gersz, Alan Howell, Renee Kirk, Malgorzata Gil‐Moore, Crystal L Mallow, Amber Foster, Anton P Porsteinsson, Andrew Feigin, Maurice Zauderer

**Affiliations:** ^1^ Vaccinex, Inc., Rochester, NY USA; ^2^ Vaccinex, Inc, Rochester, NY USA; ^3^ Alzheimer’s Disease Care, Research and Education Program (AD‐CARE), Rochester, NY USA; ^4^ NYU Langone Health, New York, NY USA

## Abstract

**Background:**

Semaphorin 4D (SEMA4D, CD100) and its receptors Plexin B1, B2 have been identified in multiple transcriptomic/genomic studies as a key signaling pathway associated with reactive gliosis and disease risk in Alzheimer’s Disease (AD), Huntington’s Disease (HD), Multiple Sclerosis, and vascular dementia. Our lab has reported that SEMA4D protein is upregulated in diseased neurons and activates astrocytes via receptor binding, resulting in downregulation of metabolic transporters and release of inflammatory cytokines. In a completed randomized Phase 2 study in HD (NCT02481674), SEMA4D blocking antibody pepinemab prevented metabolic decline in glucose uptake (FDG‐PET) reduced plasma GFAP, biomarkers of astrogliosis, and slowed cognitive decline employing multiple cognitive scales. Mechanistic and clinical studies investigate pepinemab effects on changes associated with reactive gliosis, neuroinflammation, and cognitive decline.

**Method:**

Effects of SEMA4D blockade on neuropathology, behavior, and vascular integrity were evaluated in a mouse model of HD (Hu97/18) and an in vitro brain chip model. In the phase 1b/2 SIGNAL‐AD trial (NCT04381468), 50 individuals with mild AD dementia (MMSE 17‐26) were treated for 12 months with pepinemab (40 mg/kg) or placebo, Q4W, IV infusion. Key objectives included safety, cognition, and biomarker assessments.

**Result:**

In preclinical disease models, SEMA4D blocking antibody reduced astrocyte and microglial activation markers, increased synaptic markers, and improved deficits in spatial learning and memory. In a human brain chip model, pepinemab restored α‐synuclein‐induced disruption of vascular integrity. Evaluation of amyloid‐induced damage in the brain chip model is ongoing. In SIGNAL‐AD, pepinemab was well‐tolerated and meaningful improvements in cognitive assessments were observed in the MCI‐early AD subgroup (>70% slowing of cognitive decline in CDR‐SB, iADRS, and ADAS‐Cog13). O‐link proteomic analysis of CSF revealed treatment‐related reductions in biomarkers associated with reactive astrocytes (GFAP), microglia clearance (LILRB4, MARCO), and tau pathology (GAP‐43 and SNAP25).

**Conclusion:**

Given the many physiological parallels between glial activation and inflammatory processes in HD and AD, results from these studies suggest that preventing astrocyte activation and reducing brain inflammation and associated vascular disruption with pepinemab treatment could be an attractive alternative or complement to anti‐Aβ antibodies and support the broad application of glial regulators to treat cognitive dysfunction and neurodegenerative disease.